# Dietary brewer’s spent yeast enhances growth, hematological parameters, and innate immune responses at reducing fishmeal concentration in the diet of climbing perch, *Anabas testudineus* fingerlings

**DOI:** 10.3389/fnut.2022.982572

**Published:** 2022-09-07

**Authors:** M. Gokulakrishnan, Rajesh Kumar, Bindu R. Pillai, S. Nanda, S. K. Bhuyan, Rakhi Kumari, Jackson Debbarma, S. Ferosekhan, G. M. Siddaiah, Jitendra Kumar Sundaray

**Affiliations:** ^1^College of Fisheries (OUAT), Brahmapur, India; ^2^ICAR-Central Institute of Freshwater Aquaculture, Bhubaneswar, India

**Keywords:** sustainable aquaculture, low-cost protein, innate immunity, nutrient retention, carbon footprint

## Abstract

A 60-day feeding trial was conducted to optimally reduce the fishmeal level in climbing perch (*Anabas testudineus*) fingerling diet using a dietary brewer’s spent yeast biomass (BSY) based diet. In this study, five isonitrogenous (35% CP) and isocaloric (19.15 MJ/Kg) feeds were prepared by replacing 0 (BSY0), 25% (BSY25), 50% (BSY50), 75% (BSY75) and 100% (BSY100) of fishmeal protein using BSY protein. A total of 225 numbers of uniform-sized climbing perch fingerlings (3.29 ± 0.09 g) were randomly stocked in the 15 rectangular FRP (Fiber-reinforced plastic) tanks (150 L capacity). The experimental fish were fed twice daily at 4% BW for the first fortnight and later reduced to 3% BW based on satiation. At the end of the feeding trial, the weight gain (WG) of fish increased with the increasing BSY incorporation rates corresponding to fishmeal content and peaked at 77.88%, and beyond that, WG decreased. Food conversion ratios decreased as dietary BSY levels increased and peaked at 76.28%. All other growth and feed utilization parameters followed a similar trend of weight gain. Hepatosomatic index (HSI) and viscerosomatic index (VSI), A:G ratio, serum catalase activity, and monocytes were unaffected and the total serum protein, albumin, globulin, alanine aminotransferase (ALT), aspartate aminotransferase (AST), respiratory burst activity, lysozyme levels, myeloperoxidase activity, hemoglobulin, red blood cells, white blood cells, neutrophils, eosinophils, lymphocytes, and gut protease activities were increased significantly (*P* < 0.05) with the increasing replacement levels and peaked between 25 and 75%. The serum SOD activity and total platelets were decreased, whereas the serum uric acid and gut amylase activities were increased significantly to the increasing levels of FM replacement in the diets (*P* < 0.05). Among treatments, the BSY100 resulted in an overall poor growth response combined with relatively reduced values in nearly all biochemical parameters. The whole-body composition was nearly unaffected. The integrated biomarker response of various biochemical indicators from the different treatments has shown that the 50% fishmeal protein can be optimally replaced by BSY, which would cause an 18% reduction in the Economic conversion ratio (ECR) and −270.28 gCO_2_e^–^ reduction in carbon footprint value per kg of climbing perch fingerlings production.

## Introduction

Ensuring food security for an estimated 9.7 billion population in the year 2050 is a primary goal for the food production sectors of the world (1). In that, aquaculture remains the fastest growing sector with an average annual growth rate of 5.3% and produces around 82.1 million tons of fish every year (2). In addition, aquaculture is a most efficient feed to protein converter (30%) coupled with a reduced carbon footprint than any other animal producing process (3). Sustainable aquafeed production is pivotal for supporting future aquaculture production to provide nutritious fish protein to the future population. However, the continuous growth of aquaculture production in the last decade restricted the protein resources available for aquafeed production. Marine ingredients are finite sources, and their utilization in aquafeed production in recent times is being reduced by plant-based alternative sources and their protein concentrates (4). Nevertheless, using plant-based ingredients accompanies ethical and sustainability issues including crop intensification, arable land, water and energy use and their availability for human consumption (4, 5). Hence, there is a necessity for sustainable protein ingredients for aquafeed production, such as by-products and waste biomass from other production processes, to support future aquaculture development (6).

**FIGURE 1:**
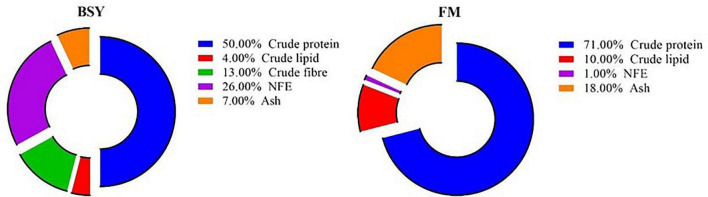
Chemical composition of fishmeal (FM) and brewer’s spent yeast (BSY).

Brewer’s spent yeast (BSY) is the agro-industrial waste biomass of *Saccharomyces* sp. from breweries worldwide. It is obtained after completing several fermentation cycles in the beer brewing process at the rate of 1.7–2.3 kg/m^3^ of beer produced (7). Disposal of this viable yeast biomass is often hazardous to the natural ecosystems posing 900 lb BOD and 600 lb of suspended solids from producing 1,000 barrels of beer (1 beer barrel = 158.99 liters) (8). On the other hand, the filtered spent yeast biomass can be deactivated by either chemical or thermal processing and utilized for nutritional purposes (9). This deactivated BSY biomass comprises excellent nutritional values and is cheaper in cost (around 0.3–0.6 USD per Kg) (10). The nutritional profile of BSY biomass is comparable with fishmeal, with around 50% crude protein (Figure 1). The essential amino acid profile of BSY biomass is almost similar to fishmeal except for arginine, lysine and methionine (11, 12). Also, BSY biomass comprises high levels of essential micronutrients like B complex vitamins and minerals (8). Being single-cell biomass, unlike other plant and animal-based proteins, BSY poses peculiar polymers (β-glucans, chitin, mannoproteins) on its cell wall capable of stimulating an innate immune response and concurrent disease resistance in fishes. Additionally, a higher amount of nucleic acid (12–20% of total N) in the BSY biomass often causes health problems in mono-gastric animals and restricts its usage in terrestrial animal feed production (13). However, fishes are ammo-telic that process nitrogen waste differently, accompanied with enhanced uricase activity, thus can efficiently utilize higher dietary BSY levels. Hence, the BSY biomass can be used as a sustainable protein source for aqua feed production, which would also increase sustainability in beer production. Moreover, previous studies have elucidated the optimal replacement rate of fishmeal with BSY in the diet of different fish species, viz., 60% in Nile tilapia, *Oreochromis niloticus* (14), 45% in Thai panga, *Pangasianodon hypophthalmus* X *Pangasius bocourti* (10), 20–30% in rainbow trout, *Oncorhynchus mykiss* (15, 16). 25% in European seabass, *Dicentrarchus labrax* (17), 47% in Asian seabass, *Lates calcarifer* (18), 50% in pacu, *Piaractus mesopotamicus* (19), 30% in gilthead seabream, *Sparus aurata* (16) and 50% in red drum, *Sciaenops ocellatus* (20).

Climbing perch, *Anabas testudineus* is an freshwater air-breathing fish native to South and Southeast Asian countries (21). Its feeding habit classified as omnivorus more inclined to carnivorous (22). It is an important food fish in its native regions because of its delicacy, appealing flavor, flesh quality and excellent nutrient profile (essential amino and fatty acids, vitamins and minerals like Fe, Cu) (23). Climbing perch is suitable for high-density intensive culture systems due to its air-breathing ability that thrives well in hypoxic water conditions (23). In recent years, the farming of climbing perch has been geared up in the Indian subcontinent after the standardization of the captive seed production technique of climbing perch in ICAR- Central Institute of Freshwater Aquaculture, Bhubaneswar, India (21). Climbing perch is cultured mostly in intensive and high densities; hence apart from the growth, their feed should also improve the health status of the fish. On the other hand, BSY is an excellent protein source, comprises numerous health-benefiting nutrients, and is cheaper than most protein sources. Hence, it is hypothesized that the BSY can be effectively utilized for climbing perch feed production. Consequently, the improved growth and health status with the reduction in the feed cost will have significant impact on the profit of climbing perch farming. Previously Mapanao et al. (24) proposed that 75% of fishmeal content (26.25 g) can be optimally replaced with black soldier fly meal (BSFM) in the diet of climbing perch, which elucidates the growth potentiality of this species to the unconventional protein source based diet. Also, previous studies using BSY in fish nutrition have shown that BSY might likely be more utilizable in omnivores than in carnivores. Considering these, an attempt was conducted to determine the optimal BSY incorporation level at reducing fishmeal concentration in the diet of climbing perch.

## Materials and methods

### Animal ethics statements

The experiment was conducted with the consent of the ethical committee of the ICAR-Central Institute of Freshwater Aquaculture, Bhubaneswar, India. All protocols involving the use of fishes in the experiment were in accordance with the CPCSEA guidelines (Committee for the Purpose of Control and Supervision of Experiments on Animals), Ministry of Environment and Forests, Government of India.

### Fish husbandry and feeding trial

Three hundred climbing perch fingerlings were obtained from the Air-breathing fish hatchery, ICAR-Central Institute of Freshwater Aquaculture, Bhubaneswar, India. The experimental fish were initially fed with a control diet (35% CP; 8% CL; 19.15 MJ kg^–1^ GE) for 15 days for acclimatization to the experimental conditions. After acclimatization, 225 numbers uniformly sized climbing perch fingerlings (mean weight 3.29 ± 0.09 g; *n* = 30) were randomly stocked in the 15 FRP tanks (150 L) at a rate of 15 fishes/tank. The treatments were assigned randomly to individual tanks as triplicates. Each tank was maintained under the natural light regime with a mild aeration supply throughout the experimental duration. During the feeding trial, the fishes were fed with their respective diets at 4% BW for the first fortnight and later reduced to 3% BW based on satiation for 45 days twice a day (10:00 and 16:00 h). The tanks were siphoned and around 20% water was replaced daily to remove fecal matter and uneaten feed particles and to maintain optimal water quality. The major water quality parameters like temperature (28.2 ± 0.45°C), dissolved oxygen (5.1 ± 0.24 mg L^–1^), pH (8.1 ± 0.11), total ammonia nitrogen TAN (<0.50 mg L^–1^) and nitrite (<0.50 mg L^–1^) were measured daily following standard methods (25) and were ranged within the optimal levels for fish culture (26).

### Experimental diets

Five isonitrogenous (35% CP) and isocaloric (19.15 MJ.kg^–1^) diets were prepared according to the nutritional requirement of climbing perch (27). The diet formulations and proximate composition are presented in Table 1. The control diet (BSY0) of climbing perch fingerlings contained 20% fishmeal. The test diets were formulated by replacing 25% (BSY25), 50% (BSY50), 75% (BSY75) and 100% (BSY100) of fishmeal protein with BSY biomass protein (48% CP as feed basis) procured from a local brewery (Pragati Enterprises Hyderabad, India). Other feed ingredients like fishmeal (64% CP as feed basis), fish oil, soybean meal, groundnut oil cake (GNOC), maize, de-oiled rice bran (DORB), vegetable oil and vitamin-premix were procured from the local market. Briefly, all ingredients were weighed and mixed homogenously in mechanical mixer and the sufficient quantity of water was added to make dough. The doughs were steam cooked for 15 min and cooled to room temperature before mixing of oil and vitamin-premix. After that, the doughs were passed through the mechanical pelletizer to obtain 2 mm sinking pellets. The pellets were dried in oven for overnight at 60°C and stored at 4°C in sealed polythene bags until use.

**TABLE 1:** Ingredients and chemical composition of experimental diets.

Diets	BSY0	BSY25	BSY50	BSY75	BSY100
**Ingredients (g.kg^–1^ as feed basis)**					
Fishmeal	200	150	100	50	0
Brewer’s spent yeast (BSY)	0	70	135	200	265
Soymeal	300	300	255	230	190
Groundnut oil cake (GNOC)	80	100	150	180	230
Maize	130	150	130	100	65
De-oiled rice bran (DORB)	240	180	180	190	200
Vitamin mineral mix¥	10	10	10	10	10
Sunflower oil	20	20	20	20	20
Fish oil	20	20	20	20	20
**Proximate composition (g.100 g^–1^ as dry basis)**					
Crude protein	35.16	34.88	35.1	35.32	35.62
Crude lipid	8.13	8.01	7.89	7.78	7.67
Crude fiber	6.46	7.02	7.49	7.84	7.86
NFE*	36.71	37.16	37.12	36.88	37.01
Ash	13.54	12.93	12.4	12.18	11.84
Gross Energy (MJ.kg^–1^)	19.15	19.06	19.11	19.19	19.27

*NFE – Nitrogen Free Extract; ¥Aqua grow-up (Anfotal nutritions, Noida, India) – Each kg contains; Vitamin A, 5,000 IU; Vitamin D3, 1,000 IU; Vitamin B1, 10 mg; Vitamin B2, 10 mg; Vitamin B6, 5 mg; Vitamin B12, 15 mcg; Vitamin B3, 75 mcg; Vitamin B5, 10 mcg; Vitamin C, 150 mg; Vitamin E, 25 mg; Vitamin H, 5 mg; Vitamin B9, 5 mg; Ca, 225 mg; Co, 20 mg; Mn, 60 mg; Fe, 30 mg; Cu, 2 mg; Zn, 2 mg; K, 20 mg; Mg, 2 mg; Choline Chloride, 50 mg.

### Sampling and analytics

After the 60-day feeding trial, fish starved for a day before final sampling. The total fish biomasses of individual tanks were weighed for evaluating growth performance. Seventy-five fishes (5 fishes/tank) were taken for whole-body composition analysis. For serum biochemical and hematological analyses, 105 (7 fish/tank) and 45 (3 fish/tank) fishes were taken, respectively. Within that, 45 fishes (3 fish/tank) were randomly selected to dissect the liver and intestine for body mass indices and digestive enzymes analyses. The fishes were euthanized by immersing in 150 ppm clove oil (Eugenol) for 10 min. for dissection and proximate analysis. The whole body and the dissected liver and intestine were immediately stored at −80°C.

### Serum and blood sample preparation

For the serum and blood collection, the fishes were anesthetized with 50 μl/L clove oil for 3 min. The blood was collected by caudal puncturing using a 23G needle and 1 ml un-heparinized syringe and allowed to clot for 30 min. The tubes were centrifuged at 2,000 *g* for 15 min. at 4°C, and the collected serum was pooled together with each replicate and stored at −20°C for further biochemical analysis. The blood samples to analyze respiratory burst activity and differential blood cell counts were collected using 2.7 % EDTA coated syringes of the same dimensions and transferred to EDTA coated vials.

### Tissue homogenate

Tissue extracts were prepared by homogenizing the liver and intestine separately with 1:9 ratio of ice-cold sucrose solution (0.25 M) and subsequently centrifuged at 6,000 *g* for 15 min. at 4°C. Then, the obtained supernatants were stored at −20°C for digestive enzyme analysis.

### Biochemical composition

Proximate analyses of feeds and whole body of fish were determined using standard methods (28). The samples were oven-dried at 105°C up to reaching constant weight. The crude protein content was measured by the Kjeldahl method (N × 6.25) (KEL PLUS, Pelican Equipments, Chennai, India), and the crude lipid and crude fiber contents were estimated by the Soxhlet method and acid/alkali digestion, respectively (SOC PLUS and FIBRA PLUS, Pelican Equipments, Chennai, India). The crude ash content was determined by incinerating the samples in the muffle furnace at 600°C for 12 h.

### Serum biochemical analysis

Antioxidants analysis like superoxide dismutase (cat. no. 706002) and catalase (cat. no. 707002) were analyzed by kit (Cayman, United States) with an ELISA plate reader (iMark plate reader, Biorad, United States), and total serum protein, serum albumin, uric acid, alkaline phosphatase, alanine aminotransferase (ALT), aspartate aminotransferase (AST), cholesterol levels were analyzed by kit (Diatek, Kolkata, India) with a UV-Vis Spectrophotometer (LI-2800, Lasany International, India).

### Immunological analysis

Serum respiratory burst activity was measured by reducing nitro-blue tetrazolium (NBT) by intracellular superoxide radicals, as mentioned by (29). The serum lysozyme activity was measured using lyophilized *Micrococcus luteus* (Sigma, United States) proposed by (30). The serum myeloperoxidase activity was calculated according to (31) using 3, 3’, 5, 5’-tetramethylbenzidine (TMB) and hydrogen peroxide.

### Hematological analysis

Hematological parameters like differential cell counts (total red blood cells, total white blood cells, neutrophils, eosinophils, basophils, monocytes, lymphocytes, total platelet cells), and hemoglobin contents were analyzed using an automatic blood cell analyzer (HOPE Diagnostics, Bhubaneswar, India).

### Digestive enzymes analysis

The digestive enzymes like α-amylase and protease activities were measured using maltose and tyrosine as standards respectively, and lipase activity was measured by kinetics using UV-Vis Spectrophotometer (LI-2800, Lasany International, India). The α-amylase (EC 3.2.1.1) activity was measured as per (32) using starch as substrate. The protease activity was measured by method proposed by Walter (33) modified by Hidalgo et al. (34). The lipase activity was measured by using sodium myristate as a substrate Faulk et al. (35). The activities of digestive enzymes were expressed in specific activities (U.mg^–1^ of protein) where one unit of activity is equal to μmol of end product generated per minute (μmol.min^–1^).

### Growth performance and calculations


Weightgain(WG,g)=Finalweight-Initialweight

Percentageweightgain(WG%)=Finalweight-InitialweightInitialweightX 100

$$\mathrm{Average}\mathrm{daily}\mathrm{weight}\mathrm{gain}\left(\mathrm{ADWG},g.{\mathrm{day}}^{-1}\right)=\frac{Finalweight-Initialweight}{\mathrm{Experimental}\mathrm{days}}$$

Specificgrowthrate(SGRg,%BWday-1)=Ln(Finalweight)-Ln(Initialweight)DoE(Days)X 100

$$\mathrm{Feed}\mathrm{conversion}\mathrm{ratio}\left(\mathrm{FCR}\right)=\frac{Dryweightoffeedgiven\left(g\right)}{Wetweightgainoffish\left(g\right)}$$

$$\mathrm{Protein}\mathrm{efficiency}\mathrm{ratio}\left(\mathrm{PER}\right)=\frac{Wetweightgainoffish\left(g\right)}{Proteinintake\left(g\right)}$$

$$\mathrm{Protein}\mathrm{production}\mathrm{value}\left(\text{PPV}\right)=\frac{\begin{array}{c} Final\left(fishweightxbodyprotein\right)\\ -Initial\left(fishweightxbodyprotein\right) \end{array}}{Proteinintake\left(g\right)}$$

Hepatosomaticindex(HSI%)=Weightofliver(g)Weightoffish(g)X 100

Viscerosomatic index (VSI%)=Weightofviscera (g)Weightoffish(g)X 100

$$\mathrm{Albumin}\mathrm{globulin}\mathrm{ratio}\left(\mathrm{AG}\right)=\frac{Serumalbuminlevel\left(g/dL\right)}{Serumglobulinlevel\left(g/dL\right)}$$

$$\mathrm{Economic}\mathrm{Conversion}\mathrm{Ratio}\left(\mathrm{US}\$/\mathrm{Kg}\mathrm{of}\mathrm{fish}\right)=\left[\frac{dietfed\left(Kg\right)}{weightgain\left(Kg\right)}\right]\;XFeedprice\left[\frac{US\$}{Kg}\right]$$

### Carbon footprint calculation

The reduction in carbon footprint value while replacing fishmeal from the climbing perch diet was estimated by computing the carbon footprint of global fishmeal production from the available literature. According to a report by Cashion et al. (36), the carbon footprint value of fishmeal reduction from 6.963 MMT of forage fish landings was about 2.0252 megatonnes CO_2_e. This value arose from the summing up of total greenhouse gas (GHG) emissions from the fuel used during the fishing phase and energy use during the processing phase of fishmeal production. The above-reported carbon footprint value (corresponds to 6.963 MMT of capture forage fisheries) has been converted for global fishmeal production [16 MMT of forage fish landings (37)] by assuming the carbon footprint of fishmeal production is more or less analogous worldwide. The computed value approximates the carbon footprint of domestic fishmeal production (from capture fisheries to fishmeal reduction) that excludes the transportation (import and export) and distribution of fishmeal. The computed carbon footprint value of global fishmeal production is approximately around 4.65 megatonnes of CO_2_e, using which the value of carbon footprint per gram of fishmeal replacement was computed at approximately about −1.212 gCO_2_e (negative value represents a reduction of carbon emission) that used as a unit value for carbon footprint calculation per kg of climbing perch production fed with different experimental diets in the present experiment.

### Integrated biomarkers response calculation

The integration of various physiological biomarkers of climbing perch fingerlings was carried out as per the method proposed by Beliaeff and Burgeot (38) and Chen et al. (39). To do so, the values of different biomarkers were normalized (Z) using the formula,


Y=(X–m) /s

Where Y is the normalized value of the biomarker; X is the mean value of the biomarker of each treatment group; m and s are the mean and standard deviation of the biomarker respectively. Z was computed as Z = –Y or Z = +Y depending on the expected biological effect of BSY inclusion on each biomarker (’+’ and ‘–’denotes induction and inhibition respectively). At last, the biomarker score (S) was computed using the following formula,


S=Z+|Min|

Where *S* ≥ 0 and | *Min*| is the absolute value of the lowest value of each normalized biomarker.

The integrated biomarker scores of the individual biomarkers were plotted in radar graphs in ascending order for individual treatments. When S_*i*_ and S_*i*+1_ are plotted as two consecutive scores in a clockwise direction of a given radar plot, the area (A_*i*_) formed by S_*i*_ and S_*i*+1_ on the graph can be calculated as


$$A_{i}=\frac{S_{i}}{2}sin\text{β}\left(S_{i}cos\text{β}+S_{i+1}Sin\text{β}\right)$$

Where,


$$\text{β}=Arctan\left(\frac{S_{i}sin\text{α}}{S_{i}-S_{i+1}cos\text{α}}\right),\text{α}-\frac{2\pi }{n},Sn+1-S1$$

Here, n represents the number of radii in the radar plot equal to the number of biomarkers. Finally, the integrated biomarkers response (IBR) index is calculated as the total area covered by the radar plot


$$IBR=\sum_{i-1}^{n}Ai$$

### Statistical analysis

The values of all parameters in the present study are expressed in Mean ± SD. All data were checked for normality using the Kolmogorov-Smirnoff test and homogeneity of variance using Levene’s test. And the data were analyzed for significant differences using one-way ANOVA with a 95% confidence level (*P* < 0.05). In addition, the data of all dependent variables were subjected to orthogonal polynomial contrasts (linear, quadric, cubic) to assess the response trend and to fit the best model for each dependent variable (40). The adjusted *R*^2^ was calculated according to Kvalseth (41) to determine the best fit model for each dependent variable. For the variables that showed significance for cubic regression, broken line regression was performed to elucidate the response trend and optimal inclusion level (42). All statistical analyses were performed in SPSS (IBM, United States) software version 25.0, and the graphs were plotted using GraphPad Prism (Version 9.4.0) software.

## Results

### Growth, nutrient utilization and organo-somatic indices

**FIGURE 2:**
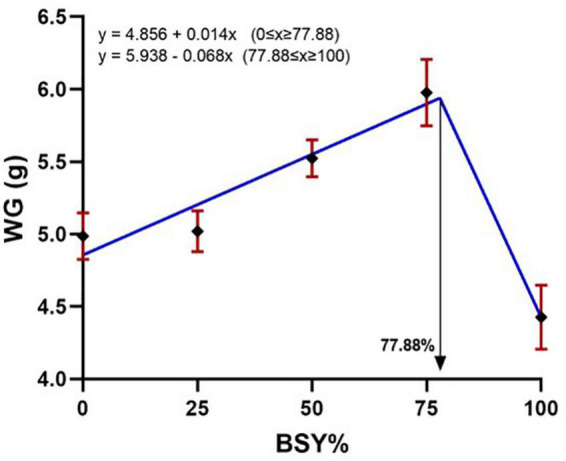
Broken line regression model of individual weight gain (WG) of climbing perch fingerlings to the BSY incorporated diet for 60 days. The X-axis represents the gradient inclusion levels of BSY in the place of fishmeal. The values are expressed as Mean ± SD, *n* = 3.

**FIGURE 3:**
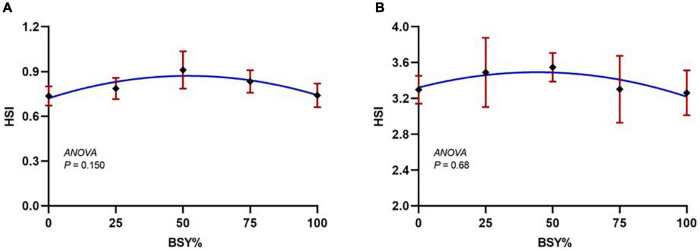
The responses of organo-somatic indices of climbing perch fingerlings to the graded incorporation level of BSY in diet for 60 days. The X-axis represents the gradient inclusion levels of BSY in the place of fishmeal. **(A)** HSI, Hepatosomatic index and **(B)** VSI, Viscerosomatic index. The values are expressed as Mean ± SD, *n* = 3.

The climbing perch fingerlings exhibited a good acceptance rate toward their respective test diets during the 60-day feeding trial. The overall growth performance and feed utilization of experimental fishes to the graded levels of BSY incorporated diets are presented in Table 2. The growth and feed utilization parameters, such as final body weight, individual weight gain, average daily weight gain, specific growth rate, protein conversion ratio and protein product value were significantly increased (*P* < 0.05) with the increasing brewer’s spent yeast incorporation rates corresponding to fishmeal content and peaked around 75%, above which the growth and feed utilization were decreased. The feed conversion ratio displayed a similar but opposite trend toward dietary BSY incorporation in the climbing perch fingerlings. All growth and nutrient utilization parameters were best explicated by the cubic order polynomial (COP) model (*P* < 0.05) against the dietary BSY incorporation; hence broken line regression model was adopted to elucidate the optimal BSY incorporation level in the climbing perch fingerling diet for each growth and nutrient utilization parameter (Table 2). The broken line regression model of individual weight gain against dietary BSY incorporation suggests that the statistically highest individual weight gain could be achieved at 77.88% reduction of fishmeal by dietary BSY incorporation in the climbing perch fingerling diet (Figure 2). The relationship between the dietary BSY incorporation to the organo-somatic indices, including hepatosomatic index (HSI) and viscerosomatic index (VSI), are given in Figures 3A,B. The mean values of HSI and VSI showed no significant differences (*P* > 0.05) among the treatments in the present experiment.

**TABLE 2:** Growth performance and feed utilization of climbing perch fingerlings to the BSY incorporated diets.

Parameters	Treatments					ANOVA	Orthogonal polynomial regression			
	BSY0	BSY25	BSY50	BSY75	BSY100	*P-value*	Model	Adj. *R*^2^	*P-value*	Opt. inc.
Initial weight (g)	3.26± 0.07	3.25 ± 0.09	3.26± 0.11	3.34 ± 0.1	3.3 ± 0.07	0.684	NR	-	-	-
Final weight (g)	8.24 ± 0.23	8.27 ± 0.19	8.78 ± 0.23	9.32 ± 0.29	7.72 ± 0.27	< 0.001	COP	0.817	< 0.001	78.48%
Weight gain (g)	4.99 ± 0.16	5.02 ± 0.14	5.52 ± 0.13	5.98 ± 0.23	4.43± 0.22	< 0.001	COP	0.882	< 0.001	77.88%
ADWG (g.day^–1^)	0.083 ± 0.003	0.084 ± 0.003	0.092 ± 0.002	0.1 ± 0.004	0.074 ± 0.004	< 0.001	COP	0.889	< 0.001	75.46%
Weight gain %	153 ± 2.84	154 ± 4.57	169 ± 3.45	179 ± 6.05	134 ± 5.33	< 0.001	COP	0.916	< 0.001	76.56%
SGR (% BW.day^–1^)	1.55 ± 0.02	1.55 ± 0.03	1.65 ± 0.03	1.71 ± 0.04	1.42 ± 0.04	< 0.001	COP	0.922	< 0.001	76.28%
FCR	2.03 ± 0.07	2.01 ± 0.06	1.83 ± 0.04	1.69 ± 0.07	2.28 ± 0.11	< 0.001	COP	0.878	< 0.001	76.28%
PER	1.55 ± 0.05	1.56 ± 0.04	1.72 ± 0.04	1.86 ± 0.07	1.36 ± 0.07	< 0.001	COP	0.889	< 0.001	76.18%
PPV	0.127 ± 0.007	0.21 ± 0.006	0.239 ± 0.006	0.269 ± 0.01	0.176 ± 0.008	< 0.001	COP	0.927	< 0.001	77.28%
Survival (%)	100	100	100	100	100	-	NR	-	-	-

ADWG, Average daily weight gain (g.day^–1^); SGR, Specific growth rate (%.day^–1^); FCR, Feed conversion ratio; PER, Protein efficiency ratio; PPV, Protein production value; NR, No relationship; COP, Cubic order polynomial trend; Adj. *R*^2^, Adjusted R square; Opt. inc., Optimal inclusion percentage of BSY.

### Biochemical indices

**FIGURE 4:**
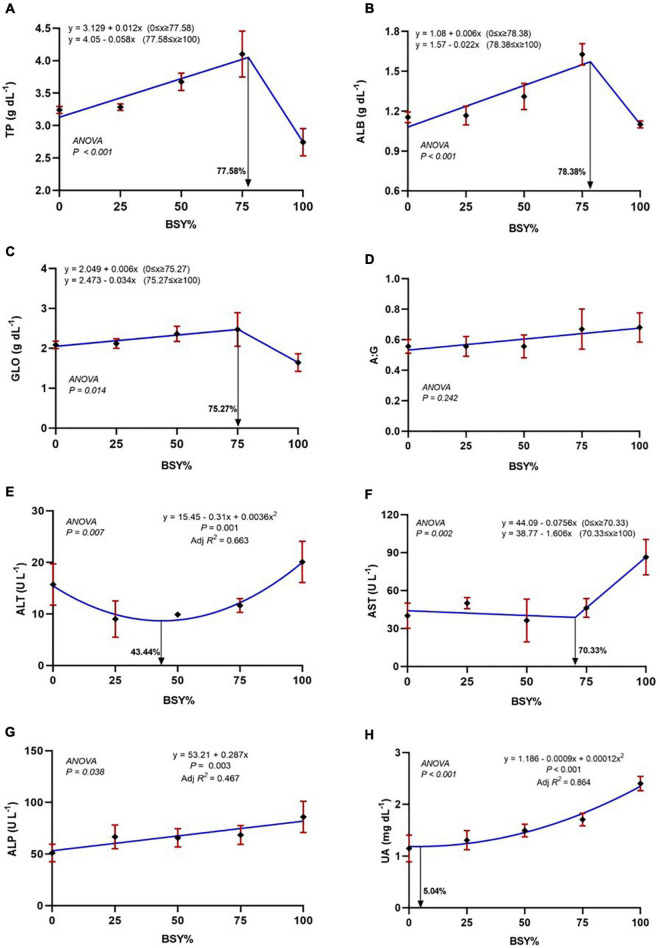
The responses of serum biochemical parameters of climbing perch fingerlings fed with BSY incorporated diet for 60 days. The X-axis represents the gradient inclusion levels of BSY in the place of fishmeal. **(A)** TP, Total protein (g dL^–1^); **(B)** ALB, Albumin (g dL^–1^); **(C)** GLO, Globulin (g dL^–1^); **(D)** A:G ratio **(E)** ALT, Alanine aminotransferase (U L^–1^); **(F)** AST, Aspartate aminotransferase (U L^–1^); **(G)** ALP, Alkaline phosphatase (U L^–1^) and **(H)** UA, Uric acid (mg dL^–1^). The values are expressed as Mean ± SD, *n* = 3.

The relationship between serum biochemical parameters of climbing perch fingerlings against the dietary BSY incorporation was presented in Figures 4A–H. The total serum protein, serum albumin and globulin levels were significantly increased (*P* < 0.05) with the increasing dietary BSY incorporation rates corresponding to fishmeal content and peaked at 77.58%, 78.38% and 75.2% respectively, above which they found decreased; whereas the A:G ratio were unaffected (*P* > 0.05) by the BSY incorporation. The response of serum total protein, albumin and globulin levels against BSY incorporation levels were best explicated by the cubic order polynomial (COP) model (*P* < 0.05); hence broken line regression was adopted to elucidate the optimal BSY incorporation level. The response of serum alanine aminotransferase (ALP) and serum aspartate aminotransferase (AST) levels showed quadratic (QOP) and cubic (COP) trends against dietary BSY incorporation respectively, and were found to have the lowest at 43.44% and 70.33% replacement levels respectively. A positive linear trend was observed between BSY incorporation level and the serum alkaline phosphatase (ALP) activities in the experimental fishes that were found highest at 100% and lowest at 0% dietary BSY incorporation in the place of fishmeal. The serum uric acid levels were found to increase with increasing levels of BSY inclusion in the diet and is best explicated by the quadratic order polynomial (QOP) model (*P* < 0.05). The QOP regression suggests that up to 5.04% fishmeal reduction by BSY incorporation did not affect the serum uric acid levels in the climbing perch fingerlings, and beyond that, it was found to be increased.

### Antioxidant and immunological parameters

**FIGURE 5:**
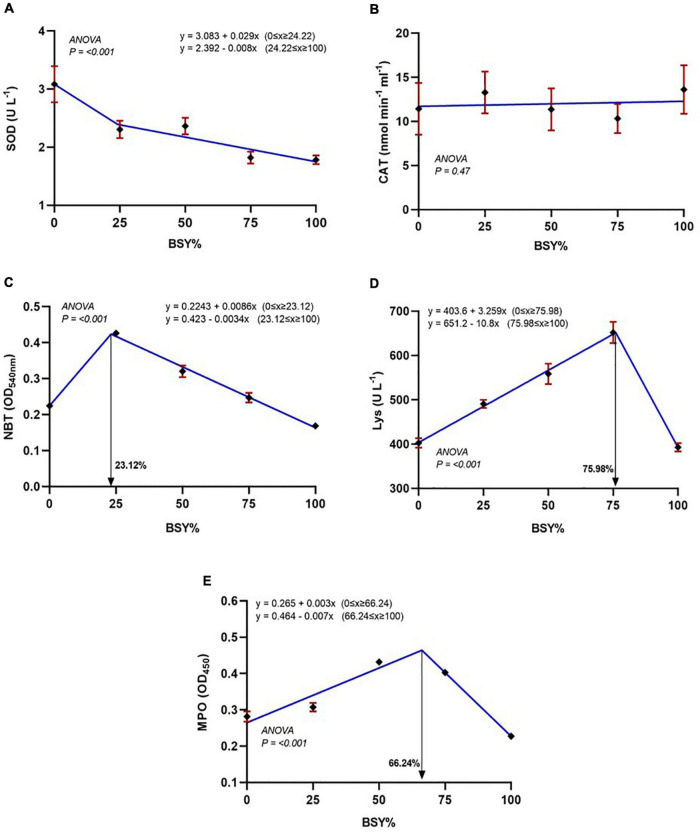
Serum antioxidant and Immunological responses of climbing perch fingerlings fed with BSY incorporated diet for 60 days. The X-axis represents the gradient inclusion levels of BSY in the place of fishmeal. **(A)** SOD, Superoxide dismutase (U L^–1^); **(B)** CAT, Catalase (nmol min^–1^.ml^–1^); **(C)** NBT, Respiratory burst activity (OD_540nm_); **(D)** Lys, Lysozyme levels (U L^–1^) and **(E)** MPO, Myeloperoxidase (OD_450nm_). The values are expressed as Mean ± SD, *n* = 3.

The relationship between serum antioxidant and immunological parameters, including SOD, catalase, respiratory burst activity, and lysozyme and myeloperoxidase levels against the dietary BSY incorporation levels in the place of fishmeal, are presented in Figures 5A–E. The serum SOD levels were significantly decreasing in cubic order polynomial (COP) trend with the increasing levels of BSY in the diet, whereas catalase showed no significant changes (*P* > 0.05). The responses of serum innate immunological parameters like respiratory burst activity (NBT), lysozyme and myeloperoxidase activities were best explicated by the cubic order polynomial (COP) model (*P* < 0.05); hence broken line regression was adopted to elucidate the optimal BSY incorporation level. These innate immunological parameters were significantly increased with the increasing levels of BSY in the diet and peaked at 23.12%, 75.98% and 66.24% of BSY incorporation in the place of fishmeal respectively.

### Hematological response

**FIGURE 6:**
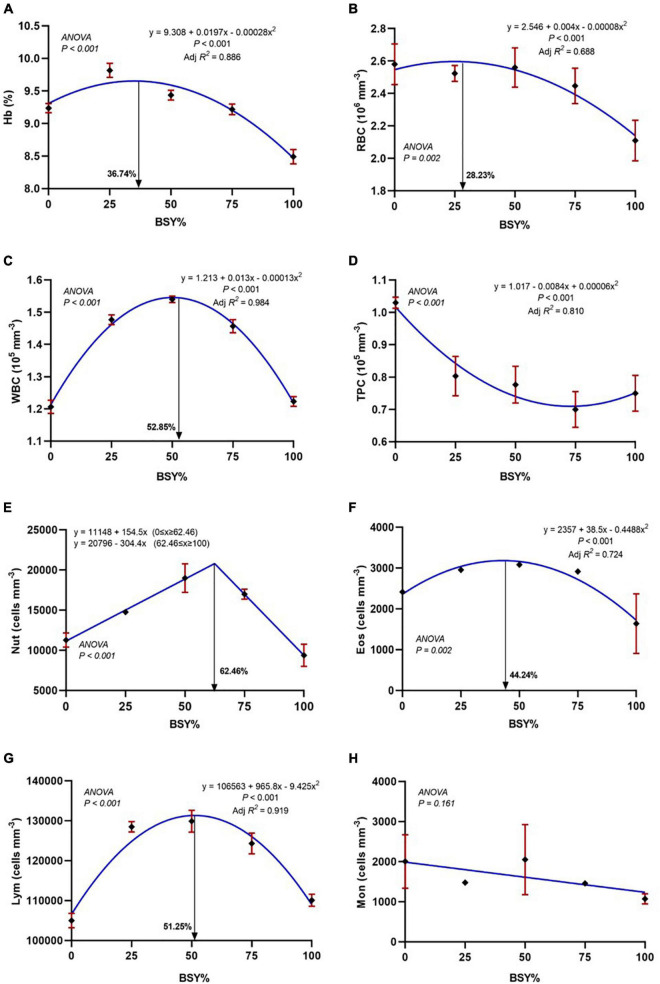
Hematological responses of climbing perch fingerlings fed with BSY incorporated diet for 60 days. The X-axis represents the gradient inclusion levels of BSY in the place of fishmeal. **(A)** Hb, Hemoglobin (%); **(B)** RBC, Red blood cell count (10^6^ mm^–3^); **(C)** WBC, White blood cell count (10^5^ mm^–3^); **(D)** TPC, Total platelets count (10^5^ mm^–3^); **(E)** Nut, Neutrophils (cells mm^–3^); **(F)** Eos, Eosinophils (cells mm^–3^); **(G)** Lym, Lymphocytes (cells mm^–3^) and **(H)** Mon, Monocytes (cells mm^–3^). The values are expressed as Mean ± SD, *n* = 3.

The relationship between hemoglobin content and differential blood cell counts of experimental fish to the graded BSY levels in the diet is presented in Figures 6A–H. The responses of hemoglobin (Hb), red blood cells (RBC), white blood cells (WBC), eosinophils and lymphocytes counts showed significance in the quadratic order polynomial (QOP) trend. They were found to be increased with the increasing levels of BSY incorporation and peaked at 36.74%, 28.23%, 52.85%, 44.24% and 51.25% of BSY incorporation in the place of fishmeal, respectively. The neutrophils count showed significance in the cubic order polynomial (COP) response to the dietary BSY incorporation levels. The total platelet count decreased with the increasing levels of BSY in the diet, and the response was significant in the quadratic order polynomial (QOP) model. The eosinophils showed no significant difference among treatments, and the basophils were found below thousand cells per cubic millimeter (<1%) in the blood of climbing perch fingerlings from all treatment groups.

### Digestive enzymes

**FIGURE 7:**
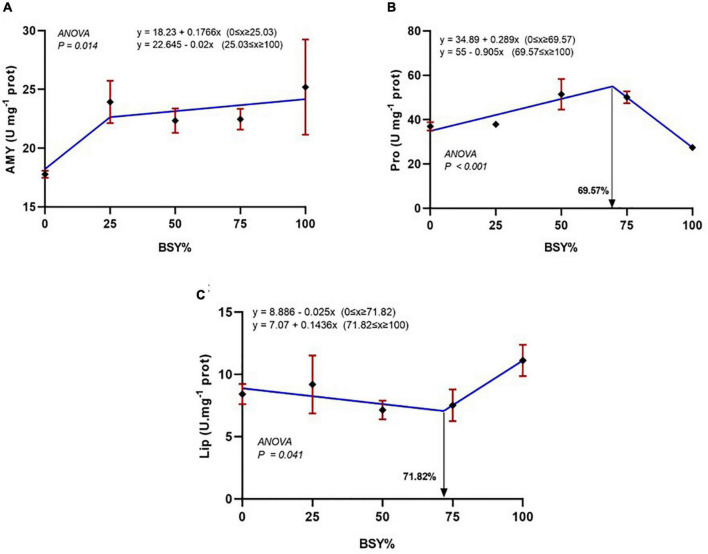
The responses of digestive enzymes activities of climbing perch fingerlings fed with BSY incorporated diet for 60 days. The X-axis represents the gradient inclusion levels of BSY in the place of fishmeal. **(A)** Amy, amylase (U mg^–1^ of protein); **(B)** Pro, Protease (U mg^–1^ of protein); **(C)** Lip, Lipase (U mg^–1^ of protein), The values are expressed as Mean ± SD, *n* = 3.

The responses of digestive enzyme activities, including amylase, protease and lipase, to the graded levels of BSY incorporation in the diet are presented in Figures 7A–C. The α-amylase activities of climbing perch fingerlings were significantly increased with the BSY incorporation levels in a cubic order trend. The total protease activities also increased significantly with the BSY inclusion level and were best explicated by the cubic order polynomial (COP) model (*P* < 0.05). The gut protease activity was found to be peaked at 69.57% of BSY incorporation in the place of fishmeal in climbing perch fingerlings’ diet. However, the reduction trend in cubic order (COP) was observed in gut lipase activities up to 71.82% of fishmeal reduction using dietary BSY; beyond that, the gut lipase activity started increasing.

### Whole-body composition

The whole-body proximate compositions of climbing perch fingerlings from different feeding groups are presented in Table 3. The moisture content of the whole body of the experimental fish did not vary significantly among the treatment groups. Though crude protein and crude lipid contents were numerically differed among feeding groups, showed no significance statistically. Also, no significant differences were observed in nitrogen-free extract and total ash contents of climbing perch fingerlings at the end of feeding trial.

**TABLE 3:** Whole-body chemical composition (%) of climbing perch fingerlings to the BSY incorporated diets for 60 days.

	Treatments						*P-value*
	Initial day	BSY0	BSY25	BSY50	BSY75	BSY100	
Moisture (%)	77.63 ± 1.32	74.89 ± 0.27	75.75 ± 0.95	75.63 ± 0.97	75.94 ± 1.71	75.78 ± 0.64	0.747
Crude protein (%)	10.09 ± 0.28	12.49 ± 0.38	12.09 ± 0.36	12.5 ± 0.5	12.9 ± 0.8	11.76 ± 0.36	0.143
Crude lipid (%)	5.87 ± 0.46	7.54 ± 0.66	6.95 ± 0.45	6.28 ± 0.4	6.22 ± 0.69	7.47 ± 0.35	0.076
NFE* (%)	2.05 ± 0.3	1.14 ± 0.13	1.69 ± 0.41	1.88 ± 0.26	1.35 ± 0.35	1.34 ± 0.28	0.077
Ash (%)	4.36 ± 0.31	3.95 ± 0.19	3.52 ± 0.21	3.71 ± 0.19	3.58 ± 0.11	3.64 ± 0.28	0.178

*NFE, Nitrogen Free Extract.

### Integrated biomarker response index

**FIGURE 8:**
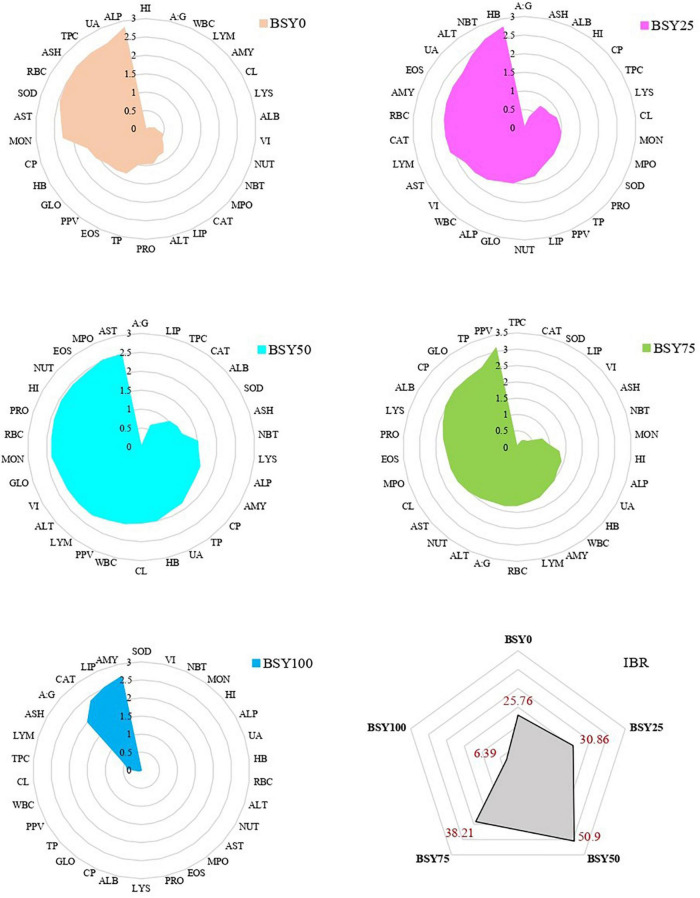
Integrated biomarkers responses (IBR) of climbing perch fingerlings fed with BSY incorporated diet for 60 days.

The integrated biomarker response for biochemical (TP, ALB, GLO, A:G, ALT, AST, ALP, UA) antioxidant (SOD, CAT) immunological (NBT, LYS, MPO) hematological (HB, RBC, WBC, TPC, NUT, EOS, LYM, MON) organo-somatic (HI, VI) and body composition and protein deposition (PRO, LIP, ASH, PPV) of climbing perch fingerlings for each treatments groups are presented in Figure 8. The IBR scores of different treatment groups were varied considerably, with the highest in BSY50 (50.9) followed by BSY75 (38.21), BSY25 (30.86) and BSY0 (25.76). The 100% fishmeal replaced group (BSY100) resulted in the lowest IBR score (6.39) among all treatment groups.

### Feed formulation cost, ECR, carbon footprint comparison

The cost comparison of different experiment feeds, and their corresponding economic conversion ratios (ECR) and carbon footprint reduction value are presented in Table 4. The prices of different experimental feeds were considerably reduced with increasing levels of fishmeal reduction in the diet from 0.79 US$/kg (BSY0) to 0.62 US$/kg (BSY100). The corresponding ECR values were found lowest in BSY75 (1.13 US$/kg of fish produced) followed by BSY50 (1.30 US$/kg of fish produced) BSY25 (1.53 US$/kg of fish produced). Interestingly, due to meager feed price, the 100% fishmeal replaced diet resulted in lower ECR (1.41 US$/kg of fish produced) than the control diet (1.60 US$/kg of fish produced) though having a poor growth response. The carbon footprint values were reduced drastically while increasing the FM replacement levels in the climbing perch diet. The BSY100 resulted in highest reduction in carbon emission (approx. −492.07 gCO_2_e^–^) followed by BSY75 (approx. −424.2 gCO_2_e^–^), BSY50 (approx. −270.28 gCO_2_e^–^) and BSY25 (approx. −127.26 gCO_2_e^–^) compared to control diet (BSY0).

**TABLE 4:** Feed formulation cost, Economic conversion ratio and carbon footprint reduction value of different experimental diets.

Ingredients	Price US$/kg	BSY0	BSY25	BSY50	BSY75	BSY100
Fishmeal	1.49	0.30	0.22	0.15	0.07	0.00
Brewer’s spent yeast (BSY)	0.54	0.00	0.04	0.07	0.11	0.14
Soymeal	0.81	0.24	0.24	0.21	0.19	0.15
Groundnut oil cake (GNOC)	0.74	0.06	0.07	0.11	0.13	0.17
Maize	0.34	0.04	0.05	0.04	0.03	0.02
De-oiled rice bran (DORB)	0.20	0.05	0.04	0.04	0.04	0.04
Vitamin mineral Premix	1.69	0.02	0.02	0.02	0.02	0.02
Veg oil	1.49	0.03	0.03	0.03	0.03	0.03
Fish oil	2.17	0.04	0.04	0.04	0.04	0.04
Price of feed		0.79	0.76	0.71	0.67	0.62
FCR		2.03	2.01	1.83	1.69	2.28
ECR (Feed cost in US$/kg of fish)		1.60	1.53	1.30	1.13	1.41
FM reduction (g.kg^–1^ of fish produced)		Nil	105	223	350	406
Carbon footprint reduction approx. (gCO_2_e^–^.kg^–1^ of fish)		Nil	−127.26	−270.28	−424.2	492.07

(Cost conversion rate: 1 US$ = 73.9 INR; Carbon emission conversion value: 1 g FM = 1.212 gCO_2_e^–^).

## Discussion

The brewers spent yeast (BSY) is one of the potential protein sources in fish nutrition that can replace a substantial quantity of fishmeal in aqua feeds (9, 16). However, the optimal inclusion level of BSY varies considerably among different fish species. Also, no previous study investigated the efficacy of BSY in the climbing perch diet. In the present study, the dietary inclusion of BSY (up to 200 g.kg^–1^ feed) in the place of fishmeal increased the overall growth performance and nutrient utilization in climbing perch fingerlings. Similar results have been documented in previous studies where the optimal fishmeal replacement levels using BSY were 60% in Nile tilapia, *Oreochromis niloticus* (14), 45% in Thai panga, *Pangasianodon hypophthalmus* X *Pangasius bocourti* (10), 20–30% in rainbow trout, *Oncorhynchus mykiss* (15, 16). 25% in European seabass, *Dicentrarchus labrax* (17), 47% in Asian seabass, *Lates calcarifer* (18), 50% in pacu, *Piaractus mesopotamicus* (19), 30% in gilthead seabream, *Sparus aurata* (16) and 50% in red drum, *Sciaenops ocellatus* (20). The BSY biomass has an excellent amino acid profile almost similar to the fishmeal except for arginine and methionine (11, 12). The precise requirements of individual amino acids for climbing perch are largely unknown, and the available amino acids in the diet of up to BSY75 seems satisfying the requirements of the climbing perch fingerlings, thus unaffected the growth. The enhanced growth responses in 50% and 75% replacement diets might be due to enriched levels of nucleic acids in the BSY biomass (43). Dietary nucleic acids are proved to be involved in feed attraction and growth enhancement in fishes (44). Besides that, dietary BSY provides essential compounds like pyruvate, amino acids and vitamins inside the fish gut that positively encourages the growth and activity of autochthonous microflora, especially lactic acid bacteria in the fish gut (45). These enhanced gut microbial activity possibly increases the microbial degradation of indigestible complex organic compounds and subsequently releases intact nutrients, and increases the nutritional values of the ingested feed, thus enhancing the growth and nutritional status of the fish. Other functional properties of BSY like immunostimulation and stress reduction could reduce the stress load of the experimental fish, and the energy conserved might be diverted into the tissue synthesis instead for homeostasis. However, the poor growth performance and feed utilization observed in 100% FM replaced diet may be because of the absence of limiting amino acids (arginine and methionine) and fishmeal associated growth promotors in the BSY100 (46). Similar results have been documented in climbing perch juveniles fed with a black soldier fly meal (BSFM) where up to 75% inclusion level resulted in enhanced growth performance but caused detrimental effect when the fishmeal was removed entirely from the diet (24). Though the 75% fishmeal replacement level resulted in significantly higher weight gain of fish in the present experiment, the broken line regression curve shows its peak at 77.88%, which indicates that dietary fishmeal content can optimally be reduced up to 155.76 g.kg^–1^ in the climbing perch fingerlings diet using BSY.

The examination of serum biochemical parameters is a reliable way to assess the nutritional and health status of fish (47). Serum proteins are essential for maintaining the osmotic balance of blood inside the vascular space (48). The increment of total serum protein levels in up-to 75% replacement level indicate the effective synthesis and metabolism of protein in the liver. Similar results have also been observed in the juveniles of pacu, *Piaractus mesopotamicus* fed with BSY incorporated diet for 54 days (19). The albumins and globulins are major class proteins present in the serum, and any changes in their levels reflect in the total serum protein content of the experimental animal. The elevated levels of serum albumins in the 50% and 75% replacement group indicate improved osmoregulation combined with efficient transportation and supply of nutrients to the various tissues to favor growth (49). Major globulins present in the serum are immunoglobulins responsible for disease resistance in fishes (48). The increasing trend of globulins indicates an elevated cellular synthesis of immunoglobulins by the lymphocyte cells of fish, which might be because of the immunostimulatory effect of BSY biomass (10). The significant decrease of aforementioned serum constituents in BSY100 may be because of the complete elimination of fishmeal from the diet that causes reduced availability of some of the amino acids essential for protein synthesis that leads to hepatic necrosis, blood cell cirrhosis and consequent dilution of blood in the fish (47, 50). Despite changes in the concentrations of different serum proteins, the ratio between albumin and globulin (AG) was consistent in experimental fishes and did not vary significantly to the different diets. Alanine aminotransferase (ALT) and aspartate aminotransferase (AST) are involved in amino acid metabolism in the hepatocytes. In case of any injury or damage in the hepatocytes, these enzymes are leaked out, and their activities are increased in the serum (51, 52). In the present study, the replacement of fishmeal using BSY biomass did not deviate serum ALT and AST activities for up to 75% replacement, and in fact, the serum ALT levels were reversed in moderate BSY inclusion rate. The presence of antioxidant peptides in BSY biomass might have reduced the lipid peroxidation of the liver that possibly reversed the serum ALT activities of the climbing perch fingerlings (47, 53). The serum alkaline phosphatase (ALP) activities of different experimental groups also followed a similar trend of ALT and AST. The significantly increased ALT, AST and ALP activities in BSY100 clearly indicate liver damage common in single-cell protein based nutrition. The serum uric acid levels were increased considerably with the increasing levels of BSY inclusion in the diet of climbing perch fingerlings. As a microbial protein source, BSY is always accompanied by higher nucleotide contents in its biomass (54). In the present study, the serum uric acid levels correlate with the dietary nucleotide intake of climbing perch fingerlings.

The aerobic life lies on the “oxygen paradox,” where oxygen, as an essential compound for life, also causes damage to tissue in its radical form (55). Like in all animals, fish also pose antioxidant defense enzymes like superoxide dismutase (SOD) and catalase (CAT), scavenging the free radicals, primarily reactive oxygen species (ROS) that are produced in the body (56). The SOD converts superoxide radicals (O_2_^–^) into hydrogen peroxide (H_2_O_2_), and it is further converted to water (H_2_O) by catalase [Xu et al., (57)]. The levels of these antioxidant enzymes in the serum imply the state of an animal’s antioxidant capacity. Inversely, in the present study, the serum SOD levels were reduced, and the catalase activity was nearly unaffected by the different diets in the present experiment. This reduction in the serum SOD levels is mainly because of the high levels of non-polar peptide fractions in the BSY biomass accompanied with vitamin C content (58), that generally associated with antioxidant properties in the biological system. The neuroactive peptide called cyclo-his-pro (CHP) with higher antioxidant properties is found plenty in BSY protein hydrolysates (59, 60). In addition, BSY biomass also comprises high levels of bio-available vitamins, minerals (Ca, P, K, Mg, Fe etc.), and free amino acids like glutamic acid, glutamine and uric acid precursor (purines) collectively support the free radical scavenging. Hence, the endogenous antioxidant enzymes requirements might be reduced in climbing perch fingerlings (58).

Immunostimulation is an effective strategy in preventing the disease occurrence in fishes, a massive threat in modern-day intensive aquaculture systems (61). In the present study, the innate immune parameters of climbing perch fingerlings, including respiratory burst activity, lysozyme content and myeloperoxidase contents, were increased in the BSY incorporated diets compared to the control. The presence of PAPMs (Pathogen Associated Molecular Patterns), a microbially conserved molecular patterns like β-glucans (50–60%), mannoproteins (35–40%) and chitins (1–3%) on the cell wall of BSY that can be recognized by the innate immune system of the fish (62). The beta-glucans from yeast cell walls potentially stimulate WBCs, significantly improve phagocytosis, lysozyme and respiratory burst activity, and eventually increase leucocyte count in fishes (63). The respiratory burst activity is a degree of superoxide anion production inside the phagocytes (64), it was doubled in BSY25 and gradually reduced with increasing levels of fishmeal replacement. Similarly, increased levels of respiratory burst activity were documented in rohu, *Labeo rohita* (65) and common carp, *Cyprinus carpio* (66). The lysozyme is an antimicrobial enzyme present in the serum, and its trend was similar to the growth performance of the climbing perch fingerlings, with the highest and lowest in BSY75, and BSY100, respectively, and the results were in agreement with the findings of Mohseni et al. (66). The myeloperoxidase is a substance with the bactericidal activity present in the phagocytic cells of animals (67), and their levels were increased in the serum of climbing perch fingerlings except in BSY100. The overall poor serum immunological response in the BSY100 group might be due to impaired nutritional balance of the feed which failed to support the basal metabolic process of the immune related protein synthesis in climbing perch fingerlings.

The hematological response of various treatments showed a positive influence of BSY inclusion in the diet of climbing perch fingerlings. The increasing trend of hemoglobin levels with unaffected red blood cell counts up to BSY75 indicates an efficient oxygen carrier system that supports active nutrient metabolism, resulting in enhanced growth response of climbing perch fingerlings. Similarly, the enhanced oxygen supply system was found in rohu, *Labeo rohita* (68), mrigal, *Cirrhinus mrigal* (69), rainbow trout, *Oncorhynchus mykiss* (70) and Nile tilapia, *O. niloticus* (71) fed with brewer’s yeast incorporated diet. These enhanced hemoglobin levels can be attributed to the considerable quantity of hematinics like B-vitamins and Iron in brewer’s yeast biomass that helps in the hematopoiesis of the fish (72). The total white blood cell counts are an indicator of disease resistance and the consequent health status of fish (61). The total white blood cells were significantly increased with the increment of BSY level in feed for upto 75%. The increment was predominantly in the neutrophils, and lymphocyte counts were primarily involved in the fish’s defense mechanism. The immunostimulants like β-glucans and chitins in the BSY cell wall have phagocytic cell receptors, and upon binding, the phagocytic cells release signal molecules called cytokines that stimulate the production of leukocytes (61). The enhanced innate immune activity in the fish serum signifies the increment of leukocytes, especially the neutrophils and lymphocytes in the climbing perch fingerlings. Similarly, enhanced leukocytes counts were documented in Thai panga, fed fishmeal replaced diet with BSY for up to 75% (10). However, the total platelets cells (TPC) were significantly reduced in all treatment groups than the control that contrasted with Thai panga, where increased platelets count levels were observed in treatment groups (10).

The digestive enzyme activities of fish mainly depend on their feeding habits (45). Being an omnivorous fish species, climbing perch has enhanced capacity to utilize dietary carbohydrates and usually have higher amylase activity in the gut (22, 45). Similarly, in the present experiment, the digestive amylase activities were almost equal to the protease activities of the climbing perch fingerlings that signify their feeding habit dependent digestive enzyme profile. The enhanced amylase activities in the test diets might be due to higher levels of complex carbohydrate compounds in the BSY biomass. Similar effects have been documented in meagre (*Argyrosomus regius*) and white seabream (*Diplodus sargus*) fed with BSY incorporate diet (45). The protease activity of fish is mostly higher than other enzymes and is usually less dependent on fish nutritional habits (34). Similarly, in our study, the protease activity was measured highest among the digestive enzymes of the gut. In addition, the increased protease activity in the 50% and 75% replacement diets correlates with their enhanced growth performances. The synergistic effects of brewer’s yeast and its subcellular compound on the host gut stimulate the production of endogenous digestive enzymes (73). Similarly, the dietary incorporated yeast-based products caused enhanced total protease activity in the rainbow trout juveniles (74). The lipase activity is highly variable in fishes (75) and in the present study, it was observed lowest among different digestive enzyme activities of the climbing perch fingerlings. The lipase activity of the fish were found increased 71.82% of replacement level that might be due to pancreatic malfunction of experimental fish against a single cell protein based diet. The proximate composition of the fish body is affected primarily by its diet and environmental conditions (76). The whole body composition, including moisture contents, was not affected significantly but improved to the increasing levels of BSY incorporation in the diet. The proximate body composition is within the normal range of climbing perch fingerlings (77). Though the difference is not significant, the increased body protein content of BSY50 and BSY75 feeding groups correlates with the enhanced total protease activity and consequent growth and feed utilization of respective group fishes. Similar enhanced body protein levels have been reported in European seabass fed with BSY incorporated diet (17).

Integrated biomarker response index (IBR) is an effective technique for determining the health state of fish during the environmental stress denoted by a single value obtained after normalizing multiple biomarkers (38). However, it is a relatively new attempt to use IBR analysis in fish nutritional studies after Chen et al. (39). The IBR indices of the present study indicated the respective health statuses of fishes from different feeding groups. Among treatments, BSY50 resulted in the highest IBR index that indicates enhanced health state of fish in BSY50 compared to all other treatment groups. Interestingly, although BSY75 resulted in highest growth and nutrient retention, the overall health indicators were found dropping during the experiment and it most probably worsens in a prolonged feeding trial. In this regard, the IBR analysis is also useful in predicting the long term effects of the feed in fishes. The enhanced health status of fishes of BSY50 signifies the optimal fishmeal replacement rate with BSY biomass in the diet of climbing perch fingerlings.

The carbon footprint of fishmeal production is a collective measurement of the total quantity of greenhouse gas emissions in its complete production process. It is measured by summing up the total emission of CO_2_, CH_4_ and N_2_O and expressed in the unit of CO_2_ equivalent (78). In the present experiment, reducing fishmeal incorporation not only improves the growth and health status of fish but also helps reduce the environmental impact of the climbing perch production process. In addition, the bio-utilization of BSY in the climbing perch diet improves sustainability in beer production. Apparently, the reduction in greenhouse gas emissions was linear with the fishmeal replacement rate in the experimental diets. When computed with FCR, the optimal fishmeal replacement using BSY (100 g. Kg^–1^ of feed) will reduce −494.6 CO_2_ eqv of carbon footprint per kg live weight of climbing perch production. Hence, the optimal incorporation of BSY in the diet contributes to enhancing sustainability and environmentally cleaner climbing perch fish production.

## Conclusion

In summary, the dietary replacement of fishmeal using brewer’s spent yeast in the diet of climbing perch fingerlings caused no adverse effects in fish for up to 75% replacement level. Moreover, 50% and 75% of dietary replacement enhanced growth and nutrient utilization in fish. Despite the highest growth and nutrient retention in BSY75 at the end of 60 days feeding trial, the integrated biomarker response scores showed that fishes of BSY50 had the best health status. The Economic conversion ratio was reduced by around 18%, with −494.6 CO_2_ eqv of carbon footprint reduction for every single kg of live climbing perch production in the best feeding group (BSY50) than the control diet. Therefore, for the long-term feeding practices, the 50% of dietary fishmeal protein (100 g of FM per kg of feed) can be optimally replaced with brewer’s spent yeast protein in the diet of climbing perch.

## Data availability statement

The original contributions presented in this study are included in the article/supplementary material, further inquiries can be directed to the corresponding author.

## Ethics statement

The animal study was reviewed and approved by the Ethical Committee of the ICAR-Central Institute of Freshwater Aquaculture, Bhubaneswar, India.

## Author contributions

MG: conceptualization, data curation, formal analysis, and writing—original draft and review and editing. RjK: conceptualization, data curation, investigation, methodology, and writing—review and editing. BP, SN, and JS: investigation, supervision, validation, and writing—review and editing. SB: investigation, supervision, and writing—review and editing. RkK: conceptualization, methodology, validation, and writing—review and editing. JD: methodology, resources, and writing—review and editing. SF: methodology, software, visualization, validation, and writing—review and editing. GS: methodology, visualization, validation, and writing—review and editing. All authors contributed to the article and approved the submitted version.
